# Shotgun Pyrosequencing Metagenomic Analyses of Dusts from Swine Confinement and Grain Facilities

**DOI:** 10.1371/journal.pone.0095578

**Published:** 2014-04-18

**Authors:** Robert J. Boissy, Debra J. Romberger, William A. Roughead, Lisa Weissenburger-Moser, Jill A. Poole, Tricia D. LeVan

**Affiliations:** 1 Veterans Nebraska Western Iowa Healthcare System, Omaha, NE, USA and University of Nebraska Medical Center, Pulmonary, Critical Care, Sleep Medicine & Allergy Division, Omaha, Nebraska, United States of America; 2 University of Nebraska Medical Center, Pulmonary, Critical Care, Sleep Medicine & Allergy Division, Omaha, Nebraska, United States of America; 3 University of Nebraska Medical Center, Department of Pathology and Microbiology, Omaha, Nebraska, United States of America; 4 University of Nebraska Medical Center, Department of Epidemiology, Omaha, Nebraska, United States of America; U. S. Salinity Lab, United States of America

## Abstract

Inhalation of agricultural dusts causes inflammatory reactions and symptoms such as headache, fever, and malaise, which can progress to chronic airway inflammation and associated diseases, e.g. asthma, chronic bronchitis, chronic obstructive pulmonary disease, and hypersensitivity pneumonitis. Although in many agricultural environments feed particles are the major constituent of these dusts, the inflammatory responses that they provoke are likely attributable to particle-associated bacteria, archaebacteria, fungi, and viruses. In this study, we performed shotgun pyrosequencing metagenomic analyses of DNA from dusts from swine confinement facilities or grain elevators, with comparisons to dusts from pet-free households. DNA sequence alignment showed that 19% or 62% of shotgun pyrosequencing metagenomic DNA sequence reads from swine facility or household dusts, respectively, were of swine or human origin, respectively. In contrast only 2% of such reads from grain elevator dust were of mammalian origin. These metagenomic shotgun reads of mammalian origin were excluded from our analyses of agricultural dust microbiota. The ten most prevalent bacterial taxa identified in swine facility compared to grain elevator or household dust were comprised of 75%, 16%, and 42% gram-positive organisms, respectively. Four of the top five swine facility dust genera were assignable (*Clostridium, Lactobacillus, Ruminococcus,* and *Eubacterium*, ranging from 4% to 19% relative abundance). The relative abundances of these four genera were lower in dust from grain elevators or pet-free households. These analyses also highlighted the predominance in swine facility dust of *Firmicutes* (70%) at the phylum level, *Clostridia* (44%) at the Class level, and *Clostridiales* at the Order level (41%). In summary, shotgun pyrosequencing metagenomic analyses of agricultural dusts show that they differ qualitatively and quantitatively at the level of microbial taxa present, and that the bioinformatic analyses used for such studies must be carefully designed to avoid the potential contribution of non-microbial DNA, e.g. from resident mammals.

## Introduction

Inhalation of swine confinement facility dust represents a major respiratory health hazard to exposed individuals. Acute symptoms frequently described are headache, fever, malaise, chest tightness, cough, and cross-shift changes in lung function [1]–[3]. Long-term exposure can lead to chronic airway inflammation, asthma, chronic bronchitis, chronic obstructive pulmonary disease (COPD), and hypersensitivity pneumonitis [1], [4]–[6]. Livestock workers, particularly swine facility workers, are at increased risk of developing chronic bronchitis, COPD, and lung function decline compared to crop workers [7].

Chronic inhalation of organic dust is implicated in respiratory disease development and severity [1], but it remains unclear which exact components in swine facility dust are responsible for the pronounced airway inflammatory reaction that it can provoke. Although swine facility dust is mainly comprised of feed particles, microorganisms or fragments from gram-positive (+) and gram-negative (−) bacteria, archaebacteria, and fungi are present. Lipopolysaccharide (LPS) or endotoxin is present in the cell wall of gram (−) bacteria and inhalation of LPS causes an intense acute airway inflammatory response [8], [9]. Although endotoxin concentrations in swine dust have been correlated with changes in lung function [10], [11], others have found a lack of correlation of endotoxin levels within swine facilities and lung function changes. Our previous work suggests that endotoxin may not be the sole component of swine dust that mediates the inflammatory reaction [12], [13], and moreover, significant roles for gram (+) bacterial components have been described. For example, swine facility dust scrubbed of endotoxin nevertheless elicits bronchial epithelial cell inflammatory cytokine release and stimulates the epithelial cell expression of the gram (+) ligand receptor Toll-like receptor (TLR)-2 *in vitro* [14]. Likewise, non-endotoxin components in swine dust appear to modulate monocyte, macrophage and dendritic cell innate immune inflammatory responses [12]. Mice deficient in TLR-2 are significantly protected, but not completely, from swine facility dust extract-induced airway inflammation [13]. Lastly, polymorphisms in the TLR-2 gene have been associated with lung dysfunction in exposed swine facility workers [15]. Collectively, these studies support the hypothesis that gram (+) microbial components may also be important in the development of swine dust-induced chronic respiratory disease.

Despite advances in our understanding of the innate immune responses attributable to the presence of gram (−) and gram (+) bacteria in the airway, the overall composition of the microbiota and possible etiological roles of specific microbes or their encoded gene products in agricultural dusts remain unclear. This information could be important to preventative and/or therapeutic strategies targeted at specific microbes. To date, culture-dependent methods are the major strategy used to describe the microbial and fungal communities in swine facility dust. Though there is a paucity of data, the most commonly cultured microorganisms in swine dust are gram (+) bacteria dominated by *Staphylococcus, Micrococcus, and Bacillus sp*. [16]. Many fungal species have also been detected; these include *Acremonium, Aspergillus, Penicillium,* and *Cladosporium* [17]–[20]. However, it is well accepted that culture-dependent methods are ineffective in characterizing complex microbial communities.

Applied gas chromatography-mass spectrometry methods have also been used to characterize complex agricultural dust samples, and results from these studies confirm the presence of endotoxin (high levels of 3-hydroxy fatty acids) and muramic acid (a chemical marker of mainly gram (+) peptidoglycans, but also gram (−) peptidoglycans) [12]. Recent studies using molecular techniques based on PCR amplification and high-throughput sequencing of bacterial 16S rRNA genes have allowed investigators to identify a wider range of microorganisms, i.e., those that could not normally be isolated using traditional culture methods [21]–[24]. When successful, this technique has the highest resolving power for analyses of taxonomic composition, but it can have limitations due to the variable copy numbers of 16S rRNA genes in different bacterial genomes, and may be subject to other artifacts due to primer bias [25], [26]. To circumvent these limitations, shotgun metagenomic sequencing using new massively parallel “next generation” DNA sequencing (NGS) technologies such as pyrosequencing on the Roche/454 Life Sciences’ platform [27] has been adopted as a useful strategy for characterizing the microbiota present in complex microbial communities, including the alignment-based annotation of individual DNA sequence reads as having been derived from the genomes of taxa in the domains *Archaea*, *Bacteria*, *Eukaryota,* or *Virus*. Shotgun metagenomic approaches can also enable the identification of microbial genes encoding biochemical and metabolic functions. Shotgun metagenomic gene function analyses complement phylogenetic profiling because ultimately one seeks to identify the gene *products* present in agricultural dust that provoke inhalation-induced airway compromise.

In this study, total DNA from dust collected from swine confinement facilities or grain elevators was extracted and used for culture-independent metagenomic comparisons by shotgun pyrosequencing. The purpose was to compare the composition of the microbiota in swine confinement facility dust and grain elevator dust, with a focus on bacterial taxonomic composition. Dust samples from pet-free households were used in comparison studies. Analyses such as these are designed to help identify candidate biomarkers for dust-type-specific respiratory pathology.

## Materials and Methods

### Dust Sample Collection, DNA Isolation, and Metagenomic Pyrosequencing

Settled surface dust was collected from two different swine confinement facilities (housing 400–600 hogs), the storage facilities at two different grain elevators, and two different pet-free domestic homes as a control. The dust samples were obtained from surfaces approximately 3–5 feet above the floor to ensure the sampled dust had been airborne and potentially inhaled by a worker. Permission was granted by the owners of the swine confinement facilities, grain elevator facilities, and households to obtain samples in an anonymous manner. Total genomic DNA was isolated by bead beating following the manufacturer’s instructions (Mo Bio, PowerSoil Kit, Carlsbad, CA), then assayed using a Nanodrop ND-1000 UV spectrophotometer (NanoDrop Technologies, Wilmington, DE). Each DNA sample (3–5 µg) was used to prepare a shotgun pyrosequencing library using a kit for this purpose (Roche Applied Science, Indianapolis, IN) according to the manufacturer’s protocol. The multiple id, barcoded template DNAs were combined in equal amounts and titrated to obtain the optimal copies per bead (3 copies per bead). Emulsion PCR and pyrosequencing were performed with the Roche/454 Life Sciences’ Lib-L (LV) and XLR70 kits, respectively. Multiplexed shotgun metagenomic DNA pyrosequencing was performed by the Core for Applied Genomics and Ecology Laboratory at the University of Nebraska, Lincoln, using a Roche/454 Life Sciences’ GS FLX Titanium instrument (Branford, CT). GS FLX Off-Instrument Software was used to de-multiplex raw pyrosequencing reads into sample-specific bins. These six shotgun pyrosequencing metagenomic read datasets are publicly available at MG-RAST [DNAdustGrainTLV2011s (4465551.3), DNAdustGrainTLV2011e (4465547.3), DNAdustSwineTLV2011f (4465549.3), DNAdustSwineTLV2011n (4465550.3), DNAdustHouseTLV2011n (4465546.3), DNAdustHouseTLV2011p (4465548.3)].

### Relevant Publicly Available Control DNA Pyrosequencing Read Datasets Derived from Swine Feces

Three bacterial 16S rRNA gene variable region amplicon pyrosequencing read datasets (SRX065852, SRX065863 and SRX065864) and three shotgun pyrosequencing metagenomic read datasets (SRX065862, SRX065867 and SRX065871) from the same study of swine feces microbiota (SRA037229) were downloaded from the NCBI’s Sequence Read Archives [28]. These six relevant publicly available datasets were used to create two MG-RAST dataset *collections* (16S and shotgun) that were analyzed as described below and which served as controls to estimate the concordance of phylogenetic profiling results (MG-RAST’s “organism abundance profiles”) between 16S rRNA gene amplicon and shotgun pyrosequencing metagenomic approaches. The three individual shotgun pyrosequencing metagenomic read datasets (SRX065862, SRX065867 and SRX065871) from the study of swine feces microbiota were also used as described below to determine if shed lumenal cells from the swine digestive tract might contribute significant amounts of swine DNA in swine feces metagenomes and hence possibly also in swine dust metagenomes.

### BLASTn Alignment-based Partitioning of Agricultural Dust Shotgun Metagenomic Reads into “Swine/Human” and “Filtered” Subsets

Unexpectedly, we found that additional bioinformatic processing steps had to be taken with dust-derived read datasets from environments with a resident mammal, i.e., swine facility (swine and human) and household (human) datasets. Each of these datasets had to be computationally partitioned into “swine/human” and “filtered” data subsets in order to maintain the original focus of this study, which was only on the latter subset, i.e., agricultural dust microbiota. Thus, post-QC reads were aligned using the *blastn* program in BLAST+ v.2.2.25 against the unmasked swine (*Sus scrofa*) draft genome sequence (ssc_ref_Sscrofa10) and/or the unmasked human genome sequence (hs_ref_GRCh37.p5). The seed size used was six nucleotides and only the best BLASTn hit per read was considered. The NCBI BLASTn program reports expect values <1e^−179^ as zero; hence zero expect values were converted to 1e^−179^ before log_10_ transformation.

The possibility remained that some reads that aligned to the swine/human genomes may align with an even lower BLASTn expect value to a known bacterial genome sequence. Thus, the 1,480 complete and 1,659 draft bacterial genome sequences that were available on the NCBI FTP site on 11/16/2011 were downloaded and formatted as a BLASTn database for a second round of alignments, using the mammalian best-hit reads as queries. Very few of these reads yielded a significant alignment to any of the available bacterial genome sequences, but in cases where such an alignment yielded a lower BLASTn expect value than was obtained with the same read’s best mammalian genome BLASTn hit, the read was re-classified and added to the “filtered” dataset. For the control read alignments against mammalian genome sequences using the swine feces shotgun metagenomic read datasets (post-QC read datasets comprised of 127,088; 427,661; and 563,638 reads for the swine feces 1, 2, and 3 datasets, respectively), the alignment workload was reduced by using an evenly sampled subset of 20,000 reads for each of these three datasets. *Except where explicitly indicated, all results reported for the swine facility and household dust-derived datasets are based on analyses of only their respective “filtered” read subsets.*

### MG-RAST Organism Abundance Profiling of *Individual* Read Datasets and Read Dataset Collections

During MG-RAST (v. 3.0) read dataset upload, the default options for quality control (QC) were selected, i.e., base-call quality filtering, read-length filtering, and de-replication of reads, but screening against a model organism genome sequence was not selected. Individual read datasets were then used for MG-RAST organism. These *individual* read dataset MG-RAST abundance profiles were then used as input for Principal Component Analysis (PCA) performed at multiple levels of the relevant classification hierarchy, as well as two-group statistical tests performed at the lowest or “leaf” level of the relevant classification hierarchy, using the “Statistical Analysis of Metagenomic Profiles” (STAMP v. 2.0) software [29].

Read dataset *collections* (e.g., swine confinement facility dust [n = 2 samples], grain elevator dust [n = 2 samples] or household dust [n = 2 samples]) were also created in MG-RAST, and these *collections* were also used for MG-RAST organism. These read dataset *collection* results were then used as input for summary histograms at all levels in the relevant classification hierarchy. Read dataset *collection* results were also used for comparisons of MG-RAST’s *organism abundance profiles* between swine feces control datasets obtained using either 16S rRNA amplicon-based or shotgun metagenomic-based approaches.

Organism abundance profiling using shotgun metagenomic read datasets was carried out using the “best hit classification” alignment procedure against the M5 non-redundant protein database (M5NR), using the following parameter values: Max. e-Value Cutoff: 1e^−5^; Min. % Identity Cutoff: 60%; Min. Alignment Length Cutoff: 50. MG-RAST’s Lowest Common Ancestor (LCA) organism abundance profiling procedure for shotgun reads did not produce profiles that could be used with STAMP, and hence the LCA results were not compared.

Organism abundance profiling using the swine feces control “16S” read dataset *collection* was carried out using the “best hit classification” alignment procedure against the Ribosomal Database Project database (RDP, University of Michigan) [30], using the following parameter values: Max. e-Value Cutoff: 1e^−5^; Min. % Identity Cutoff: 97%; Min. Alignment Length Cutoff: 50. The top 10 taxa in the RDP-based organism abundance profiles were ranked based on their relative abundances (taxon-specific abundance/total abundance). Relative abundances values for the same taxa were obtained from the organism abundance profiles carried out using the swine feces control shotgun read dataset *collection* and the M5NR-based database. Relative abundance *ratios* were then calculated as the ratio of M5NR-based relative abundance divided by RDP-based relative abundance. Perfect concordance between the RDP and M5NR organism abundance profiles would yield a relative abundance *ratio* of 1.

### Candidate Biomarker Analyses

STAMP profile files (.spf) were created from MG-RAST abundance profiles (.tsv files) that had been created as described above using *individual* shotgun metagenomic read datasets. Candidate taxonomic biomarkers were identified at different taxonomic levels using STAMP Extended Error Graphs and pair-wise statistical tests with dust type as the group field. Welch’s t-test and Welch’s inverted method were used for estimation of the difference between group mean proportions and a 95% confidence interval. A minimum difference between group mean proportions of 0.1% was used to minimize spurious results due to low read counts. Significance (*q*) values were corrected for multiple testing using Storey’s FDR (false discovery rate). A significance threshold (ceiling) was chosen that selected the top-ranked group-distinguishing taxa (typically around 10 taxa), and then these most significantly ranked taxa were ordered by *effect size* (difference in mean proportions in the two-group statistical test).

## Results

### Use of Relevant Publicly Available Data to Estimate Concordance of Phylogenetic Profiling Results between 16S rRNA Gene Amplicon and Shotgun Pyrosequencing Metagenomic Approaches

16S rRNA gene sequencing has been widely used for phylogenetic profiling of microbial communities. Alternatively, shotgun metagenomic sequence data can be used for this purpose. Both approaches have their limitations; 16S rRNA sequencing may be biased because of unequal amplification of 16S rRNA genes, whereas shotgun sequencing may not be deep enough or the phylogenetic diversity of databases like the M5NR may be inadequate to detect rare taxa in a microbial community. To determine if these two approaches give largely similar phylogenetic profiles, we conducted a comparison of these two approaches using publicly available high-quality metagenomic pyrosequencing read datasets obtained from a study of swine feces microbiota [28]. Using data from this study, we created one MG-RAST dataset *collection* from a triplicate set of shotgun metagenomic pyrosequencing reads, and a second dataset from of a triplicate set of 16S rRNA gene amplicon pyrosequencing reads. We compared the organism abundance profiles obtained using the M5NR or the Ribosomal Database Project (RDP) databases, respectively, and the MG-RAST “Best-Hit” protocol. We defined a “relative abundance *ratio*” as the *ratio* of the relative taxon abundance (RTA) values determined by the two protocols (RTA_M5NR_/RTA_RDP_), and then plotted the relative abundance ratios obtained, together with the values for RTA_RDP_, for the ten most abundant taxa at five different taxonomic levels (Phylum to Genus; Figure S1A–E). For these two types of swine feces-derived read dataset *collections* and their respective analysis methodologies, of the top ten taxa, six Phyla, four Classes, three Orders, four Families, and four Genera have relative abundance *ratios* between 0.5 to 2.0, indicating that the two analysis methodologies for characterizing microbial communities yield reasonably concordant results. We observed that when a species is rare as assessed by 16S rRNA gene amplicon sequencing, more discordance is observed.

### Alignment of Dust DNA Reads to Human and Swine Genomes

**Figure 1:**
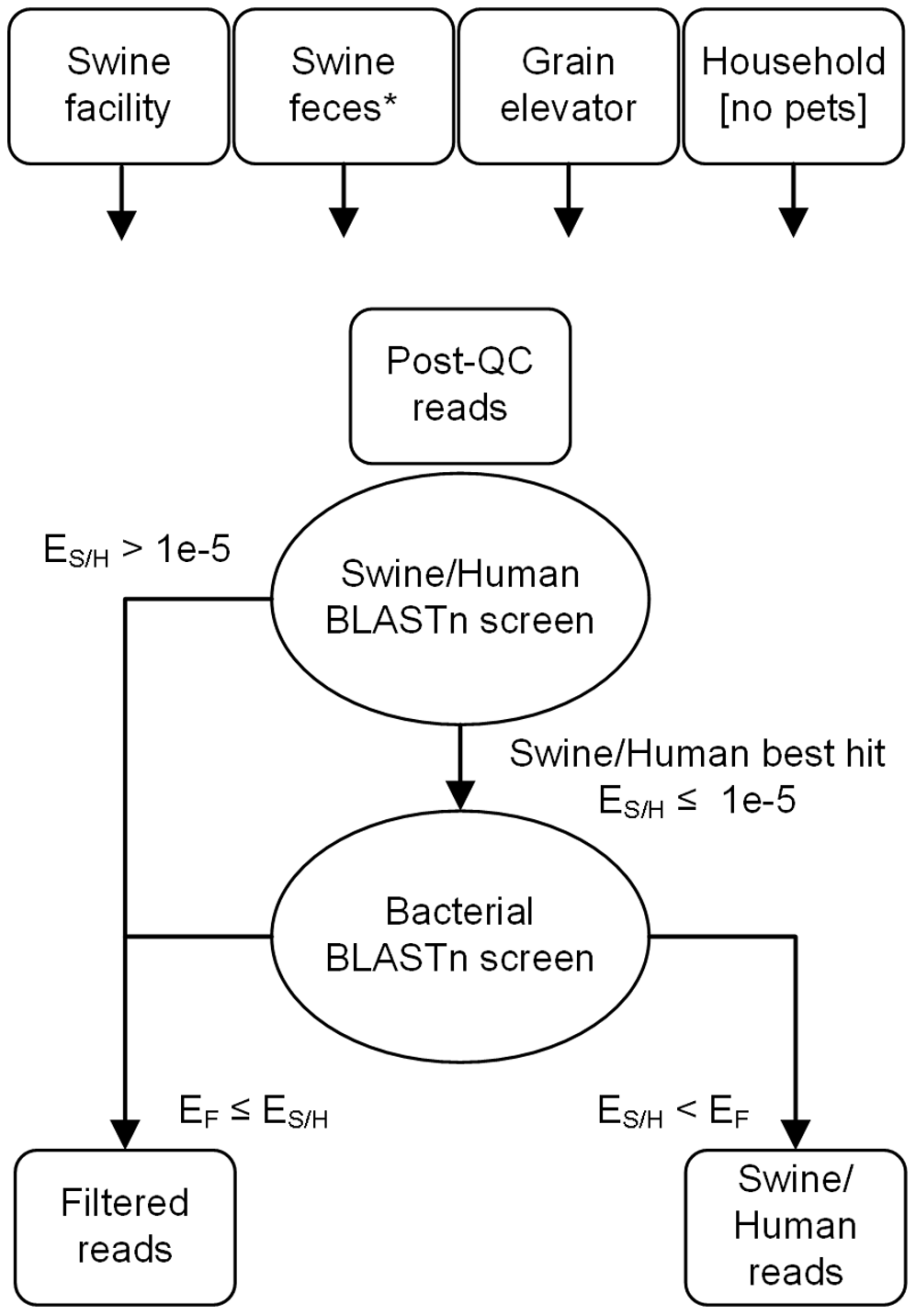
BLASTn screening scheme used for alignment of shotgun metagenomic reads to swine and human genomes. Post-QC shotgun metagenomic reads from swine facility dust, swine feces, grain elevator dust and household dust without pets were aligned against the swine draft (ssc_ref_Sscrofa10) and human genome (hs_ref_GRCh37.p5) sequence. Reads that aligned against the swine draft and/or the human reference genome sequence with an expect value of less than 10^−5^ were subsequently aligned against all finished and draft bacterial genome sequence assemblies currently available at the NCBI FTP site on 11/16/2011. Except were indicated, filtered reads were used in all subsequent bioinformatics analyses. * From the NCBI Sequence Read Archive; E_S/H_ =  expect values for reads aligned to swine and/or human genomes; E_F_ =  expect values for reads with poor alignment with swine or human genomes (filtered reads).

**Figure 2:**
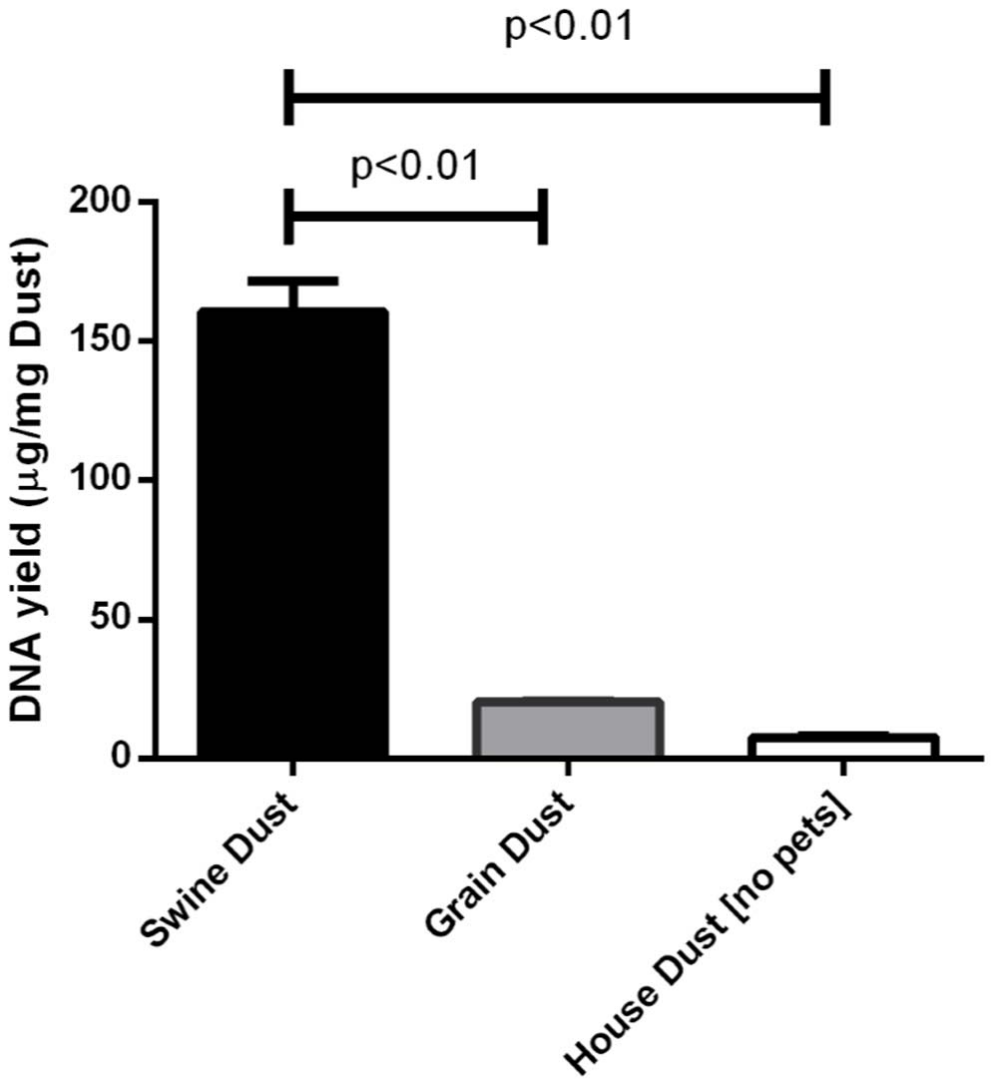
DNA yield of dust isolated from swine confinement facilities, grain elevators and households without pets. Total genomic DNA was isolated by a bead-beating protocol (Mo Bio, Power Clean, Carlsbad, CA) and quantified using a Nanodrop ND-1000 UV spectrophotometer (NanoDrop Technologies, Wilmington, DE). Each bar represents the mean DNA yield (µg/mg dust) ± SEM from two independent dust samples.

Swine facility and household datasets each had to be carefully partitioned into “swine/human” and “filtered” data subsets using a BLAST-based protocol to remove mammalian reads of swine and human origin (Figure 1), as such reads were not relevant to the focus of this study. The three different dust types (swine facility, grain elevator and household), which differ substantially in their yields of metagenomic DNA (Figure 2) and post QC metagenomic shotgun pyrosequencing reads, also differ substantially in their content of swine/human reads (Table S1). *Individual* read datasets derived from grain elevator dust or swine feces yield negligible percentages of their reads aligning to the human or swine genome (2% ±0 SEM and 2% ±1 SEM, respectively) compared to the other two dust types, which are from environments with a resident mammal, i.e., swine confinement facilities (19% ±5 SEM) and households (62% ±14 SEM). Box plots of BLAST “Expect” values show that the alignments of post-QC pyrosequencing reads to the swine or human genome are of very high quality when the query sequences are reads derived from dusts with a high resident mammalian contribution, i.e. swine and household (Figure S2). Despite the source of the swine feces control data, each of these three metagenomic shotgun read datasets can be described as having a low resident mammalian contribution and poor quality alignment to the human and swine genomes, indicating that shed lumenal cells in swine fecal debris is not the source of the mammalian DNA present in dust isolated from swine confinement facilities. Finally, except where noted, in all of our subsequent analyses using shotgun metagenomic pyrosequencing reads generated from dust samples, the “swine/human” reads from the swine facility and household dust samples were excluded as our study focus was agricultural dust microorganisms.

### Relative Abundance of Domains in Swine Facility, Grain Elevator and Household Dust “Pre-filtering.”

**Figure 3:**
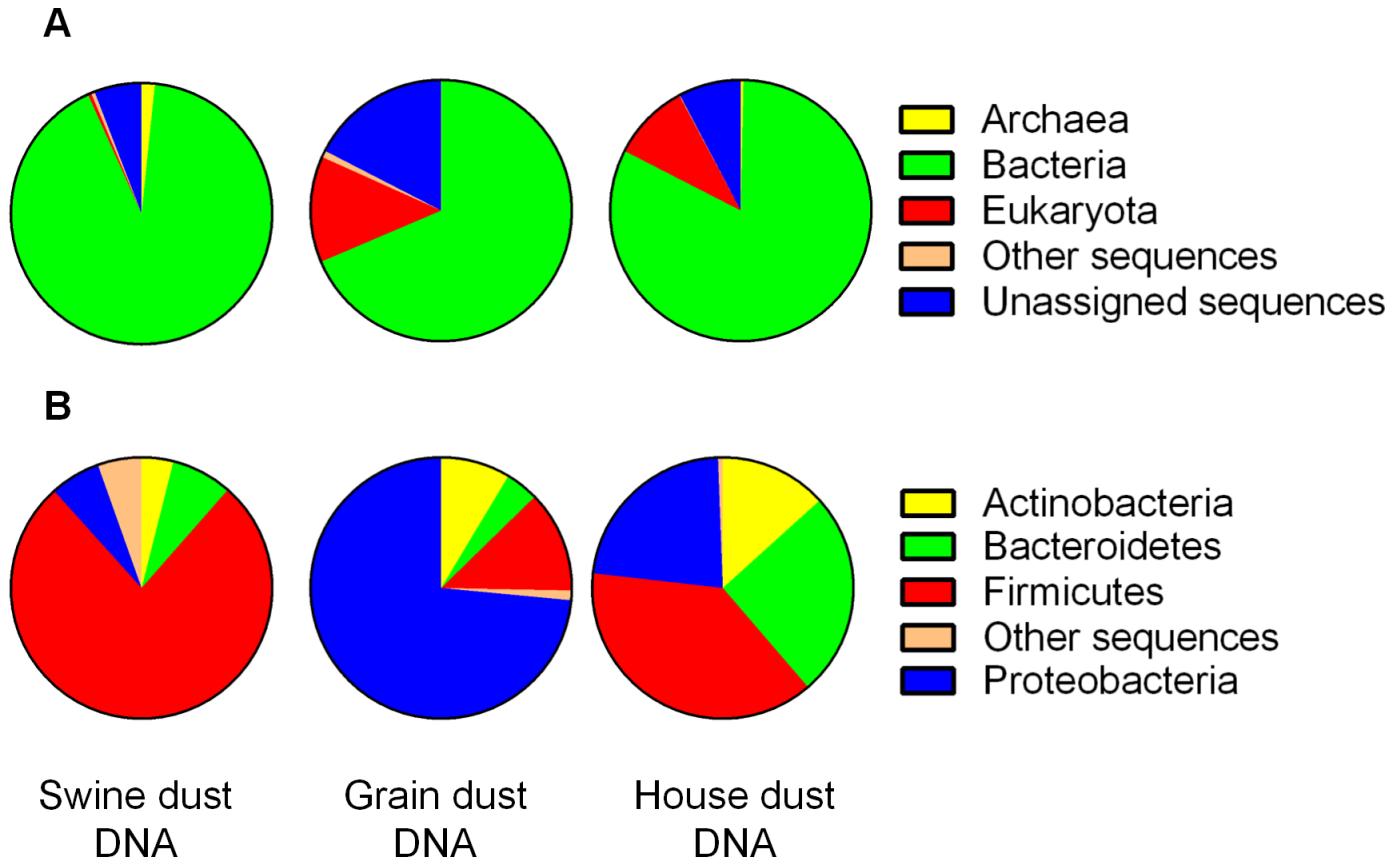
Taxonomic classification of metagenomic reads from swine confinement facility dust, grain elevator dust and household dust without pets. ***A.*** Domain level; ***B.*** Phylum level. Each pie chart represents relative abundance values expressed as the total number domains (“pre-filtered” dataset) or phyla (“filtered” dataset) from swine confinement facility dust, grain elevator dust and household dust without pets. Other sequences equals reads that align significantly to the M5NR database that are derived from taxa not listed as descendants from one of the domains; Unassigned equal reads that do not align significantly to any M5NR database sequence.

For all dust types, the domain “Bacteria” predominates with relative abundance (RA) levels of 91.6%, 68.6% and 82.2% in swine, grain and household dust, respectively (Figure 3A). Dusts from grain elevator or pet-free households have a greater RA of “Eukaryota” DNA, 13.1% and 9.6% respectively, compared to swine dust (0.4%). “Eukaryota” DNA identified was primarily from plant and fungal phyla. The predominant fungal genera identified in grain elevator dust are *Gibberlla* (0.7% RA), *Neosartorya* (0.5% RA), *Saccharomyces* (0.4% RA) *and Aspergillus* (0.3% RA), respectively, which are not found in household dust. In contrast, archaeal DNA is present in low amounts in all of the dust samples with only 1.6% RA in the swine confinement facility dust, 0.03% RA grain elevator dust and 0.3% RA in household dust. The genera *Methanosphaera* (0.3% RA), *Methanobrevibacter* (0.3%) and *Methanothermobacter* (0.2% RA) account for 50% of the archaeal DNA in swine dust. Interestingly, *Methanobrevibacter* was detected in household dust at a RA of 0.2%. Though not visible on the pie charts, the RA of viral DNA, primarily bacteriophage, in all dust types was <0.2% (Table S2).

### Relative Abundance of Bacteria “Post-filtering.”

Within the domain “Bacteria”, the top four Phyla are *Actinobacteria, Bacteroidetes, Firmicutes and Proteobacteria*, and the relative distribution of phyla are different for each dust type (Figure 3B). *Firmicutes* predominate in swine (76% RA) and household (38% RA) dust compared to *Proteobacteria* in grain elevator dust (73% RA). We also generated relative abundance profiles of the top 15 bacterial genera present in the three dust-type-specific read dataset *collections* (Figures. S3 A–C).

**Figure 4:**
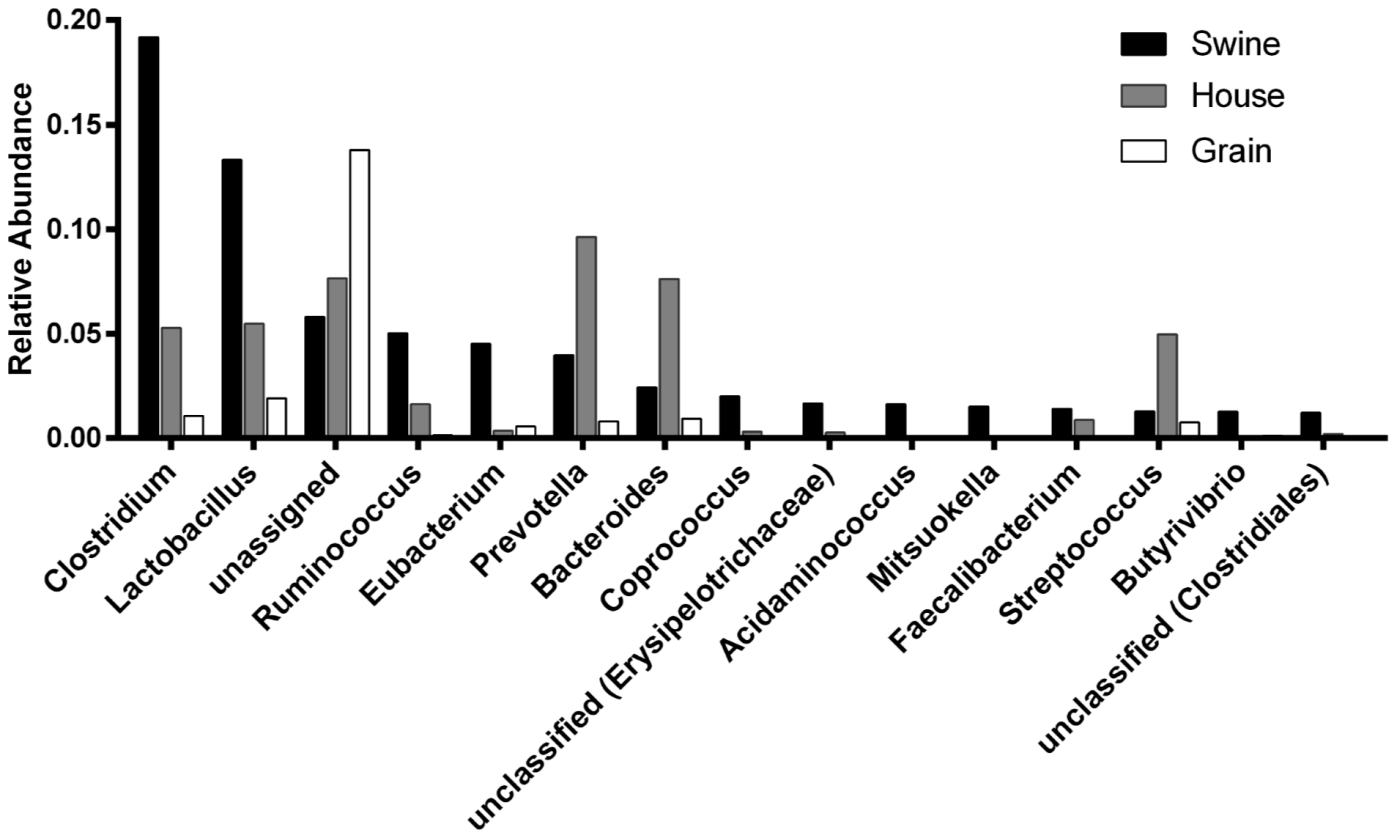
Genus abundance ranking of swine confinement facility dust reads in comparison to grain elevator dust and household dust without pets. *Relative* abundance values are expressed on the ordinate as a fraction of the total number of genera identified in swine dust. The 15 most abundant genera identified using the swine facility dust shotgun metagenomic reads and the M5NR database are shown. Relative abundance values were calculated for these same 15 genera for dust collected from grain elevators and households without pets. See Figure S4 for comparisons at the Phylum, Class, Order, and Family taxonomic levels. Black = Swine dust; Gray = House dust; White = Grain dust.

Relative abundance of genera for the swine facility dust-derived read dataset *collection* was compared to those obtained for the same genera for the other two dust-type-specific read dataset *collections* at the genus level (Figure 4). Four of the top five swine facility dust genera are assignable (*Clostridium, Lactobacillus, Ruminococcus,* and *Eubacterium*, ranging from 19% to 4% RA), and the RA levels of these four genera in grain elevator dust are all lower than values obtained for the other two dust types. In contrast, among the top 15 swine facility dust genera there are two–*Prevotella* (9% RA) and *Bacteroides* (7% RA)–that are much more significantly represented in household dust than agriculture-derived dusts. We also carried out similar analyses at the Phylum, Class, Order, and Family level (Figure S4 A–D). These analyses also highlight the predominance in swine facility dust of *Clostridia* (44% RA) at the Class level, and *Clostridiales* at the Order level (41% RA) and Clostridiaceae (20%). Although none of the assignable genera among the top 15 swine facility dust genera are more frequent in grain elevator dust, among the top 15 assignable swine facility dust phyla, *Proteobacteria* (48% RA) and *Actinobacteria* (7% RA), are more frequent in grain elevator dust than swine facility dust. At the Class level, *Gammaproteobacteria* (37% RA) are much more frequent in grain elevator dust than either of the other two dust types.

### Principal Component Analysis of Bacteria

**Figure 5:**
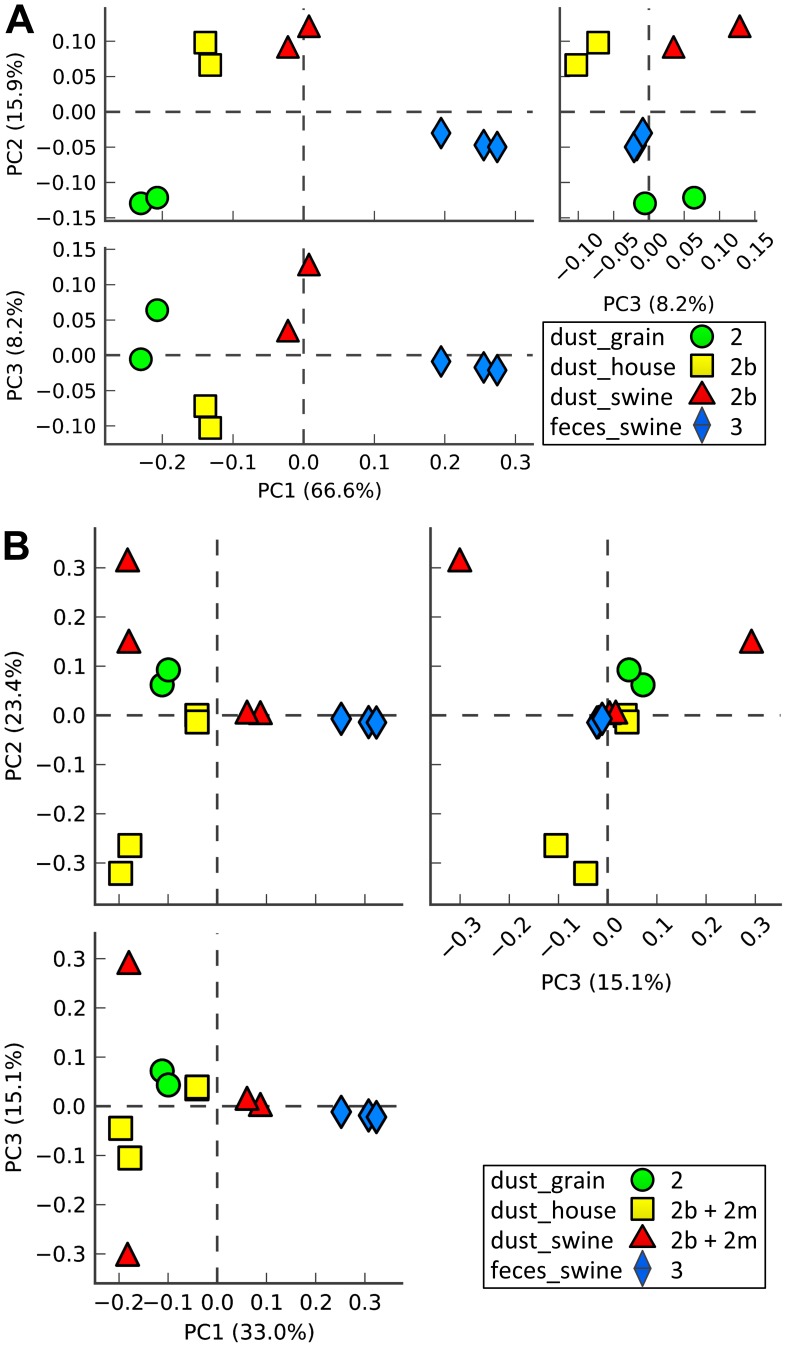
Principal Component Analysis (PCA) of shotgun metagenomic reads from swine facility dust, grain elevator dust, household dust without pets and swine feces: Relative abundance of Genera. PCA was performed in STAMP using MG-RAST Genus-level organism abundance profiles that were derived from two swine facility dust samples, two grain elevator dust samples, two household dust samples without pets and three swine feces samples. *A.* The “filtered” reads from the swine confinement facility dust and the household dust datasets were used in the analyses. *B.* The “filtered” and “swine/human” reads from the swine confinement facility dust and the household dust datasets, respectively, were used in the analyses. Each symbol represents a sample. • Grain elevator dust (green); ▪ Household dust without pets (“filtered”, yellow); ▴ Swine confinement facility dust (“filtered”, red); ♦ Swine feces (blue).

The Principal Component Analysis (PCA) plots generated using STAMP show good dust-type-specific clustering and resolution of the *individual* read datasets at all taxonomic levels, especially at the genus level (Figure 5A and Figure S5A–D). Also noteworthy is the distinguishable, separate clustering and resolution of the “swine/human” subset of reads from the “filtered” subset of reads for both the two swine facility dust individual read datasets and two household dust individual read datasets (Figure 5B). These data indicate that the individual read datasets for each sample type are more related in characteristics compared to the other sample types.

### Identification of Candidate Biomarkers in Swine Confinement Facility Dust

**Figure 6:**
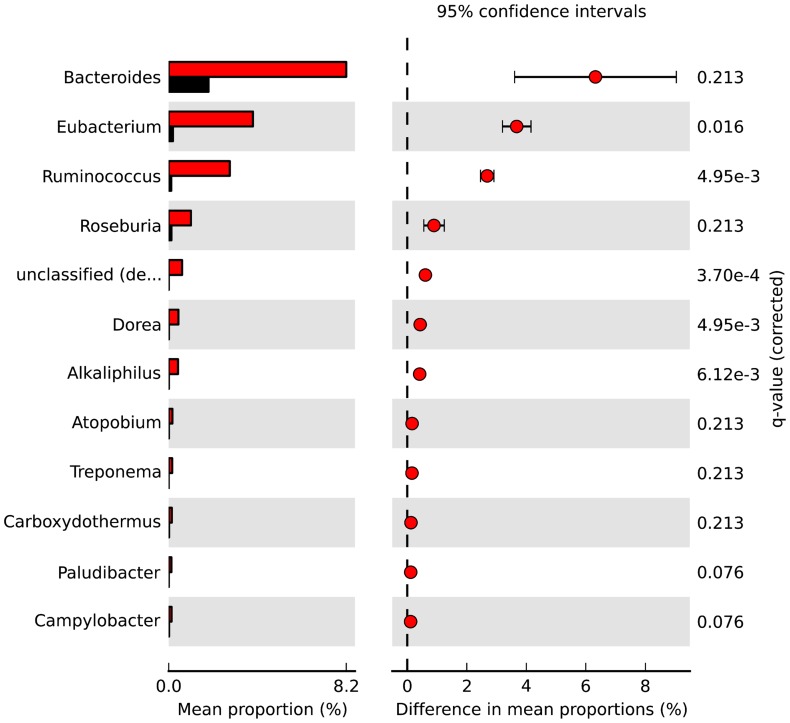
Candidate biomarker analyses based on Genus abundance profiles from dust-derived shotgun metagenomic read datasets. STAMP Extended Error Graph of the top ranked genera identified in a two-group statistical test comparing MG-RAST Genus abundance profiles generated using the M5NR database for swine facility dust dataset (red) and both household and grain elevator dust datasets (black). Ranking of the genera is based on significance (q) values, which were corrected for multiple testing and show the indicated value for Storey’s false discovery rate. The unclassified genus shown is annotated by MG-RAST as derived from *Erysipelotrichaceae*.

We also used STAMP to perform more rigorous two-group statistical comparisons (extended error graphs and bar plots based on Welch’s two-sided *t*-test) using *individual* read dataset MG-RAST organism abundance profiles to identify candidate microbial taxonomic biomarkers for swine facility dust samples compared to other dust type samples (grain elevator and household). Given the results of the PCA analyses and using these same *individual* read dataset organism abundance profiles, we focused initially at the genus level. When we compare the swine facility dust samples to the other dust samples (Figure 6), the genera *Ruminococcus* and *Eubacterium* had significant difference between group mean proportions (DP) values and low *q* values (the minimum False Discovery Rate, or FDR, at which the test is significant). This suggests that these two genera may be candidate biomarkers for swine dust compared to grain elevator or housedust without pets. The genus *Bacteroides* showed the largest DP between the two groups (swine facility dust vs. all other dust samples) though not statistically significant due to a wide confidence interval, whilst unclassified annotations that were presumed to be derived from *Erysipelotrichaceae* had the lowest *q* value, followed by the genera *Dorea, Ruminococcus, Alkalaphilus,* and *Eubacterium*.

## Discussion

This study provides an initial overview of the metagenomic constituents of dust isolated from swine confinement facilities and grain elevators using a culture-independent approach, with comparisons to those present in dusts from pet-free households. In this study after the removal of swine/human pyrosequencing reads, we found that the majority of the reads isolated from swine confinement facility, grain elevator and household dust were from the *Bacteria* domain though the distribution of phyla were dependent upon the dust type. Bacterial pyrosequencing reads from swine confinement facility dust were most commonly annotated as being derived from the genomes of *Firmicutes*, a phylum primarily composed of gram (+) microorganisms. At the genus level, the following gut bacteria predominated: *Clostridium,* an obligate anaerobe that forms endospores; *Lactobacillus*, a facultative anaerobe that degrades lactose to lactic acid; and *Ruminococcus,* an obligate anaerobe that degrades cellulose, and which is often found in ruminants. In contrast, bacterial pyrosequencing reads from *Proteobacteria*, a gram (−) phylum, predominated in grain elevator dust. The genera present in grain elevator dust were facultative anaerobes found in the gut including *Escherichia*, *Shigella*, and *Salmonella*. Collectively, these studies provide new insight into the metagenomes of complex agriculture environmental dusts, and moreover, demonstrate distinct patterns of predominate bacteria in each environmental setting.

It is now widely acknowledged that agricultural workers exposed to workplace dust are at elevated risk for airway inflammation, though the relative etiological significance of different bioactive components found within these various and complex dusts remain unclear. Prior efforts to measure the presence and significance of different microbial taxa in these dusts have primarily focused on traditional culture-dependent techniques [16]–[18], [31]–[33]. Total bacterial concentrations in swine confinement facility dust-laden ambient air have been estimated from as low as 10^4^ cfu/m^3^ to as high as 10^8^ cfu/m^3^ [18], [23], [31], [32], [34], [35]. In general, endotoxin from gram (−) bacteria, peptidoglycans from predominantly gram (+) bacteria, (1→3)-β-D-glucans, and fungal components have emerged as major pathogenic factors of concern in agricultural workplace exposure studies. Our group had previously found that both swine confinement facility and grain elevator dust contained quantitatively similar aerobic bacterial counts (10^5^ cfu/mg dust); however, qualitatively swine confinement facility dust had a higher proportion of cultured gram (+) bacteria (98%), i.e., *Staphylococcus, Bacillus, Streptomycetes* and *Enterococcus* species, compared to grain elevator dust (60% gram (+) bacteria) [36]. Our current study, which used non-traditional, culture-independent techniques, was able to provide a broader and more detailed view of agricultural dust metagenome constituents. Interestingly, our study confirmed the presence of high concentrations of bacterial DNA, and moreover, the predominance of gram (+) bacteria in swine confinement facility dusts. These results may facilitate the development of targeted strategies to prevent and/or reduce the onset and severity of inflammatory disease resulting from workplace exposures to this especially troublesome type of agricultural dust.

Indeed, our prior animal studies demonstrated that pattern recognition receptor signaling pathways–including nucleotide oligomerization domain 2 (NOD2, which senses bacterial-derived peptidoglycan); TLR-2, (which recognizes gram-positive bacterial components); and myeloid-differentiation factor 88 (MyD88, which is used by all of the TLRs except for TLR-3) –are all important in mediating airway inflammatory outcomes following exposure to swine confinement facility organic dust extracts [13], [37], [38].

In addition to finding a predominance of gram (+) bacteria in swine facility dust, we further determined that the most abundant phylum was *Firmicutes*. A recent study from Denmark using quantitative fluorescence *in situ* hybridization (FISH) with a general bacterial probe support our shotgun metagenomic sequencing results and also revealed *Firmicutes* as the dominant phylum (with *Clostridium* as the major genus) in swine confinement facility aerosols [22]. Nehme *et al.* used 16s rRNA gene amplification and denaturing gradient gel electrophoresis to survey the biodiversity of the microbiome in aerosolized swine confinement facility dust [24]. Similar to the results of the present study, 93.8% of the sequences that they obtained were related to gram (+) anaerobic bacteria and were dominated by the genus *Clostridium*. Differences in the abundance of the phylum *Firmicutes* and many genera were found to vary among swine production facility phases, i.e., farrowing and gestation buildings versus weaning and finishing buildings [21].

In addition to the characterization of the bacterial community in swine confinement facility dust compared to grain elevator dust, we detected greater percentages of DNA reads from the domain *Archaea* in swine dust compared to grain elevator dust. Archaeal DNA was only 3% of the filtered reads in swine dust and most closely aligned to the phylum *Euryarchaeota* and genera *Methanosphaera, Methanobrevibacter,* and *Methanothermobacter*, known hydrogen-utilizing methanogenic archaea. These phyla are strict anaerobes, difficult to culture, and are usually present in the intestinal tract of animals when methane production is significant, such as ruminants; thus, it is not surprising that archaea have not been cultured previously from swine confinement facility dust and comprise a small proportion of swine (non-ruminant) confinement facility dust microbiota, respectively. Nehme *et al.* reported archaea in bio-aerosols from swine confinement buildings using 16S rRNA gene amplification [23]. Despite using a different methodology, i.e., aerosolized dust and 16S rRNA amplification, they detected sequences associated with the genus *Methanosphaera stadtmanae*. Of note, the concentration of archaeal 16S rRNA gene amplicons was found to be high and comparable to those of bacteria. In contrast to Nehme *et al.*, but in concordance with the present study, a low abundance of archaea (0.3%) was detected by Kristiansen *et al.* [22]. These discrepancies could be due to differences in extraction and detection methods, as well as individual communities.

Interestingly, shotgun metagenomic pyrosequencing reads from swine facility dust and household dust (but not grain dust) yielded significant amounts of DNA of swine and human origin. Publicly available data from metagenomic studies of swine feces showed that shotgun pyrosequencing reads from this biological source contain very low levels of swine genomic DNA, suggesting that the origin of swine DNA in swine facility dust is not from swine feces. Our PCA results (Figure 5) suggest a similar conclusion based on the distinct clustering of the swine feces samples from the swine confinement facility dust samples. These results sound a significant note of caution to other metagenomic studies of dusts from environments with a resident mammal. If the focus of such studies is, like this one, on the microbiota present, then such studies should be careful to adopt a shotgun metagenomic read filtering strategy like the one described here, i.e., to exclude potentially confounding mammalian reads.

There was minimal representation of the domain *Eukaryota* in swine confinement facility dust-derived reads compared to those derived from grain elevator dust. Among the eukaryotic DNA reads, fungal species were 30-fold lower in swine dust compared to grain dust. There were no predominant fungal species in swine dust; however grain dust contained *Gibberlla*, *Neosartorya*, *Saccharomyces and Aspergillus*, which together accounted for 59% of fungal DNA. Previous studies have primarily used traditional culture-dependent techniques and thereby obtained total fungal counts that ranged in concentration from 10^3^ cfu/m^3^ to 10^6^ cfu/m^3^ in swine confinement facility and grain elevator dust. A recent study of the fungal community of swine confinement facility aerosols using amplification of small subunit rRNA found *Aspergillus-Eurotium* as the quantitatively most important fungal group [22], and these fungi have been commonly detected using culture-dependent approaches in swine confinement facility and grain elevator dust [19], [20], [31], [39].

Our results demonstrate the predominance of bacteria in all dusts studied. The predominant bacteria in swine confinement facility dust are gram (+) anaerobic bacteria from the genera *Clostridium*, *Lactobacillus* and *Ruminococcus*. This was in contrast to the predominance of gram (−) facultative anaerobes in grain elevator dust. *Ruminococcus* and *Eubacterium* were the two top bacterial taxonomic biomarkers in swine confinement facility dust compared to grain elevator dust. Further studies are needed to investigate *Ruminococcus* and *Eubacterium* and their possible role in respiratory pathology.

This study illustrates the advantages of detecting and identifying metagenomic constituents using culture-independent techniques. This metagenomic approach could next be applied to understand and determine how different feed diets, animal antibiotic use, and housing conditions affect the bacterial metagenome in various agricultural environments, which might ultimately be important in strategies aimed to minimize human disease. In addition to the caution concerning the filtering of reads derived from dusts from environments with a resident mammal, the use of larger numbers of biological replicates would enable more robust statistical estimates using, for example, software like STAMP [29] and individual read datasets in preference to broader aggregated overviews using MG-RAST read dataset *collections*.

## Supporting Information

Figure S1 **Swine feces read datatset collection taxonomic ranking and analysis methods comparison.** *Relative* abundance values for the 10 most abundant phyla (***A***), classes (***B***), order (***C***), family (***D***) and genus (***E***) were calculated based on MG-RAST organism abundance profiles that were generated using a 16S rRNA gene variable region amplicon read dataset *collection* and the RDP database (right ordinate, fraction). Results are plotted as a line. The relative abundances of the same taxa were calculated based on MG-RAST organism abundance profiles that were generated using a shotgun read dataset *collection* and the M5NR database. Relative abundance *ratios* were calculated and plotted as histograms (left ordinate, unitless).
(TIF)

Figure S2 **Expect value distribution of BLASTn alignments to the swine or human genome.** Box plots are used to summarize the distribution of the negative log_10_ of the expect values of the DNA sequence alignments of shotgun metagenomic pyrosequencing reads derived from either two swine facility dust samples, three swine feces samples, two household dust samples or two grain elevator dust samples. DNA sequences were aligned against the swine draft (ssc_ref_Sscrofa10) and human genome (hs_ref_GRCh37.p5) sequence. Lower expect values represent higher quality alignments; medians are shown by dashed lines and means by solid lines. A value of 179 is lowest negative log_10_ of expect values using the *blastn* program in the NCBI’s BLAST+ (version 2.2.25) software. Median ± range is presented. Gray = swine genome; White = Human genome.
(TIF)

Figure S3 **Relative abundance of Genera from swine facility, grain elevator, and household without pets samples.** A. Swine facility; B. Grain Elevator; C. Household with pets. read datasets. *Relative* abundance values are expressed on the ordinate as a fraction of the total number of genera identified in the specific dust sample. The relative abundances were calculated based on MG-RAST organism abundance profiles that were generated using a shotgun read dataset *collection* and the M5NR database.
(TIF)

Figure S4 **Taxonomic abundance ranking of** **swine confinement facility dust reads in comparison to grain elevator dust and household dust without pets.** *Relative* abundance values are expressed on the ordinate as a fraction of the total number of taxa identified in swine dust. ***A***. Phylum; ***B***. Class; ***C***. Order; ***D***. Family. The 15 most abundant taxa identified using the swine facility dust shotgun metagenomic reads and the M5NR database are shown. Relative abundance values were calculated for these same 15 taxa for dust collected from grain elevators and households without pets. Black = Swine dust; Gray = House dust; White = Grain dust.
(TIF)

Figure S5 **Principal Component Analysis (PCA) of shotgun metagenomic reads from swine facility dust, grain elevator dust, household dust without pets and swine feces.** PCA was performed in STAMP using MG-RAST different taxa-level organism abundance profiles (***A***. Phylum; ***B***. Class; ***C***. Order; ***D***. Family) that were derived from two swine facility dust samples, two grain elevator dust samples, and two household dust samples without pets. “Filtered” reads from the swine confinement facility dust and the household dust datasets were used in the analyses. Each symbol represents one sample. • Grain elevator dust (green); ▪ Household dust without pets (“filtered”, yellow); ▴ Swine confinement facility dust (“filtered”, red).
(TIF)

Table S1 (DOCX)

Table S2 (DOCX)
