# Supplementary figures and images for: Shotgun Pyrosequencing Metagenomic Analyses of Dusts from Swine Confinement and Grain Facilities
Source: PLoS One. 2014 Apr 18;9(4):e95578. doi: 10.1371/journal.pone.0095578 (PMC3991671; doi:10.1371/journal.pone.0095578)

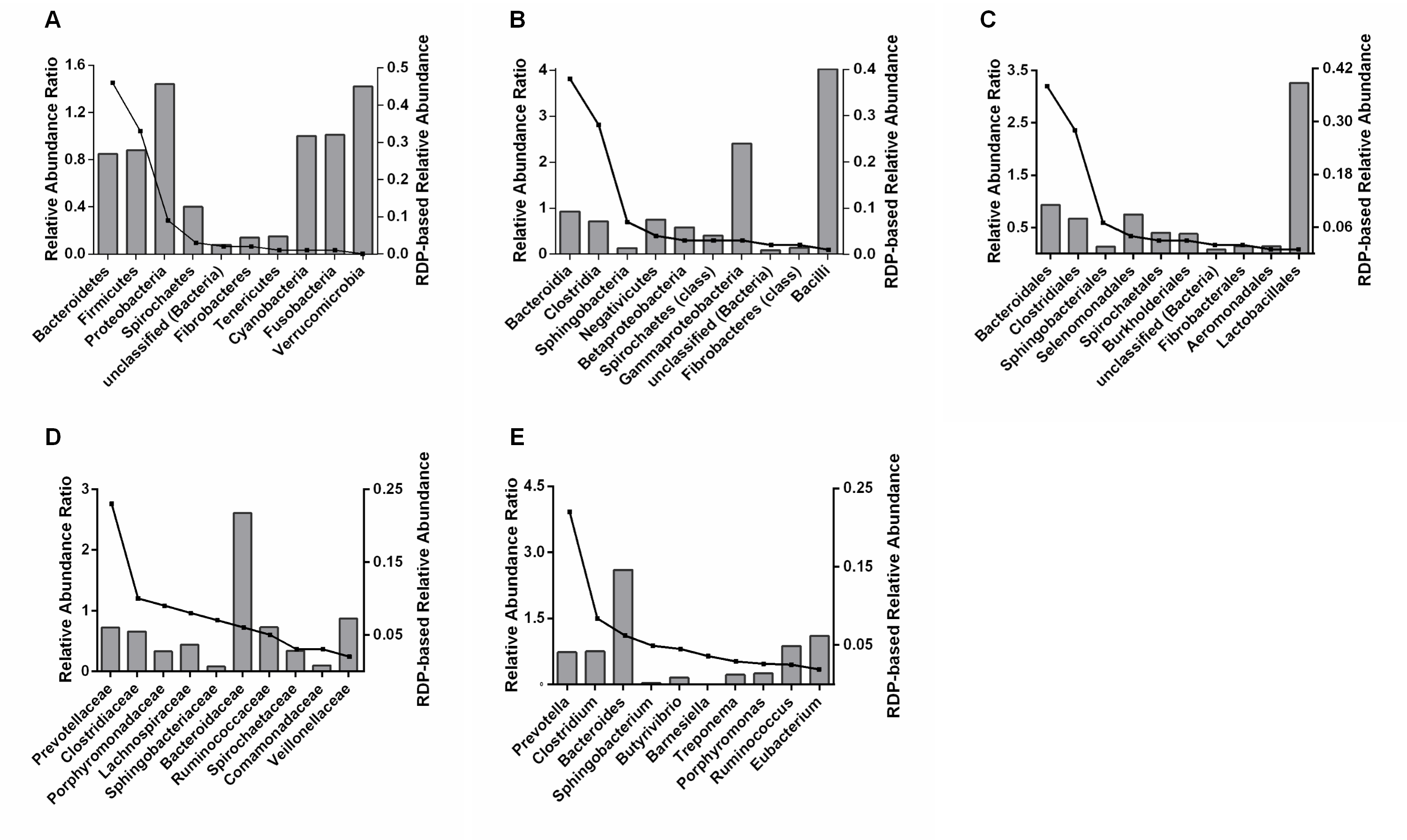

Supplement: Figure S1 — Swine feces read datatset collection taxonomic ranking and analysis methods comparison. Relative abundance values for the 10 most abundant phyla (A), classes (B), order (C), family (D) and genus (E) were calculated based on MG-RAST organism abundance profiles that were generated using a 16S rRNA gene variable region amplicon read dataset collection and the RDP database (right ordinate, fraction). Results are plotted as a line. The relative abundances of the same taxa were calculated based on MG-RAST organism abundance profiles that were generated using a shotgun read dataset collection and the M5NR database. Relative abundance ratios were calculated and plotted as histograms (left ordinate, unitless). (TIF) [file pone.0095578.s001.tif]

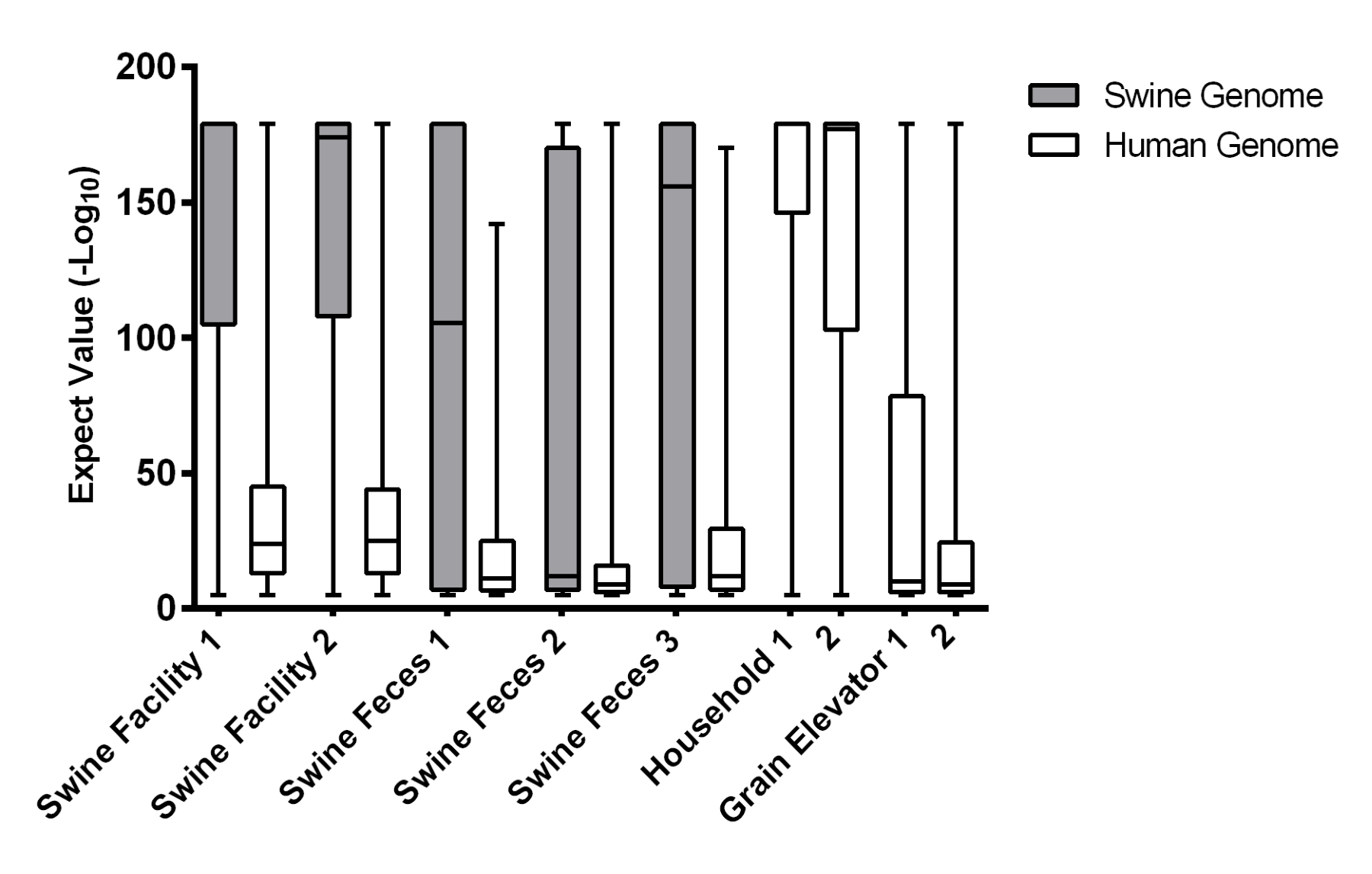

Supplement: Figure S2 — Expect value distribution of BLASTn alignments to the swine or human genome. Box plots are used to summarize the distribution of the negative log10 of the expect values of the DNA sequence alignments of shotgun metagenomic pyrosequencing reads derived from either two swine facility dust samples, three swine feces samples, two household dust samples or two grain elevator dust samples. DNA sequences were aligned against the swine draft (ssc_ref_Sscrofa10) and human genome (hs_ref_GRCh37.p5) sequence. Lower expect values represent higher quality alignments; medians are shown by dashed lines and means by solid lines. A value of 179 is lowest negative log10 of expect values using the blastn program in the NCBI’s BLAST+ (version 2.2.25) software. Median ± range is presented. Gray = swine genome; White = Human genome. (TIF) [file pone.0095578.s002.tif]

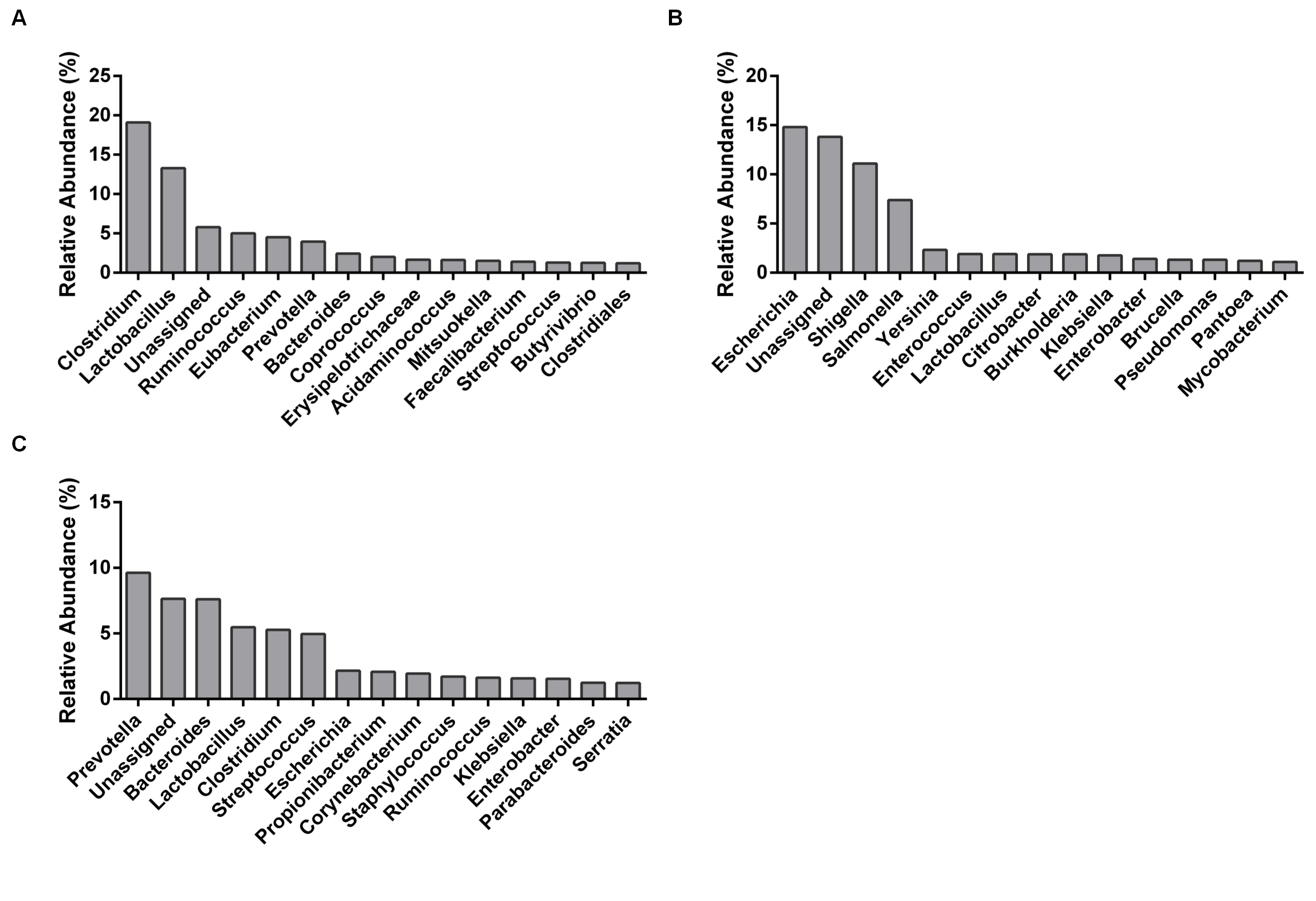

Supplement: Figure S3 — Relative abundance of Genera from swine facility, grain elevator, and household without pets samples. A. Swine facility; B. Grain Elevator; C. Household with pets. read datasets. Relative abundance values are expressed on the ordinate as a fraction of the total number of genera identified in the specific dust sample. The relative abundances were calculated based on MG-RAST organism abundance profiles that were generated using a shotgun read dataset collection and the M5NR database. (TIF) [file pone.0095578.s003.tif]

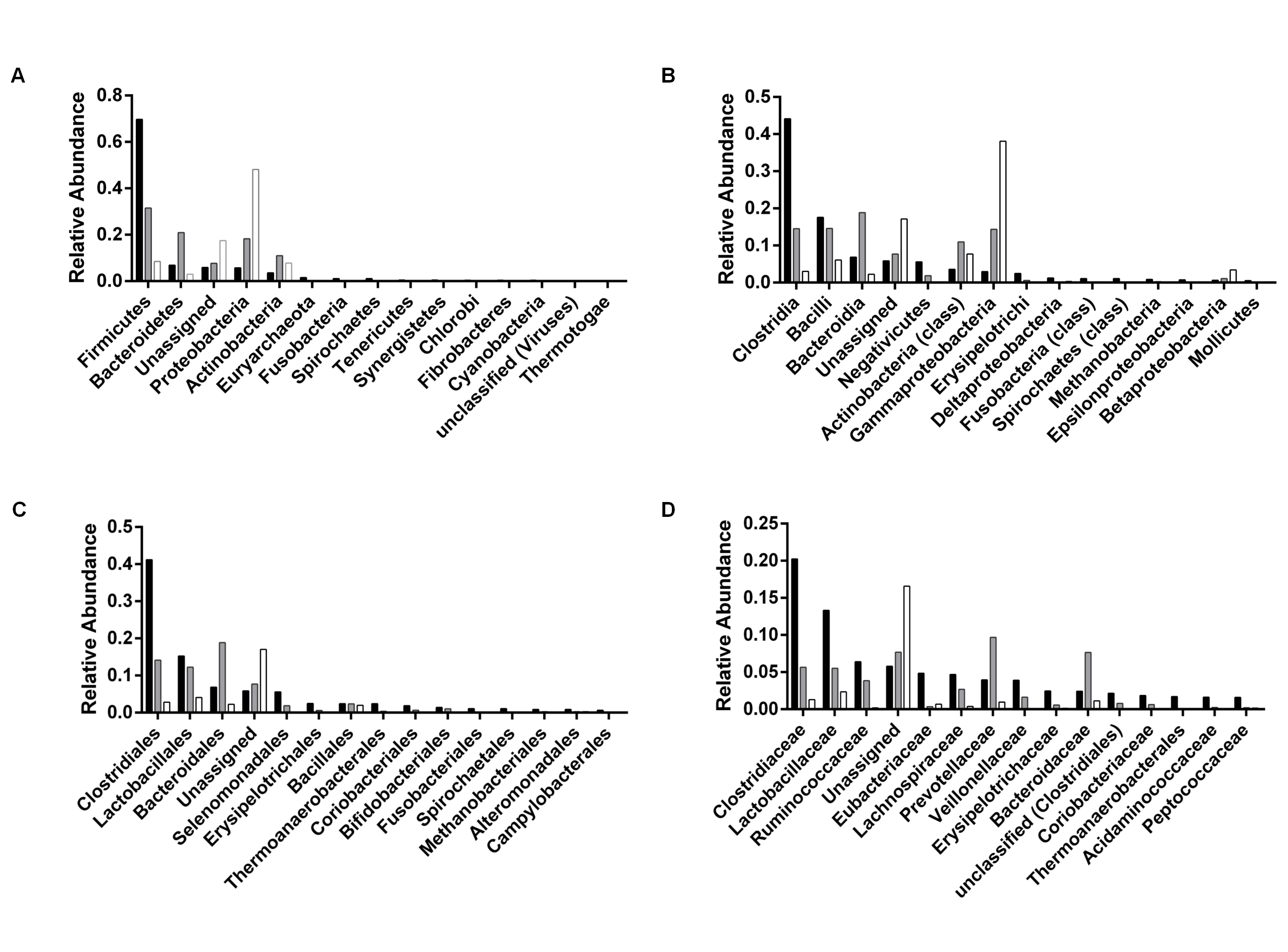

Supplement: Figure S4 — Taxonomic abundance ranking of swine confinement facility dust reads in comparison to grain elevator dust and household dust without pets. Relative abundance values are expressed on the ordinate as a fraction of the total number of taxa identified in swine dust. A. Phylum; B. Class; C. Order; D. Family. The 15 most abundant taxa identified using the swine facility dust shotgun metagenomic reads and the M5NR database are shown. Relative abundance values were calculated for these same 15 taxa for dust collected from grain elevators and households without pets. Black = Swine dust; Gray = House dust; White = Grain dust. (TIF) [file pone.0095578.s004.tif]

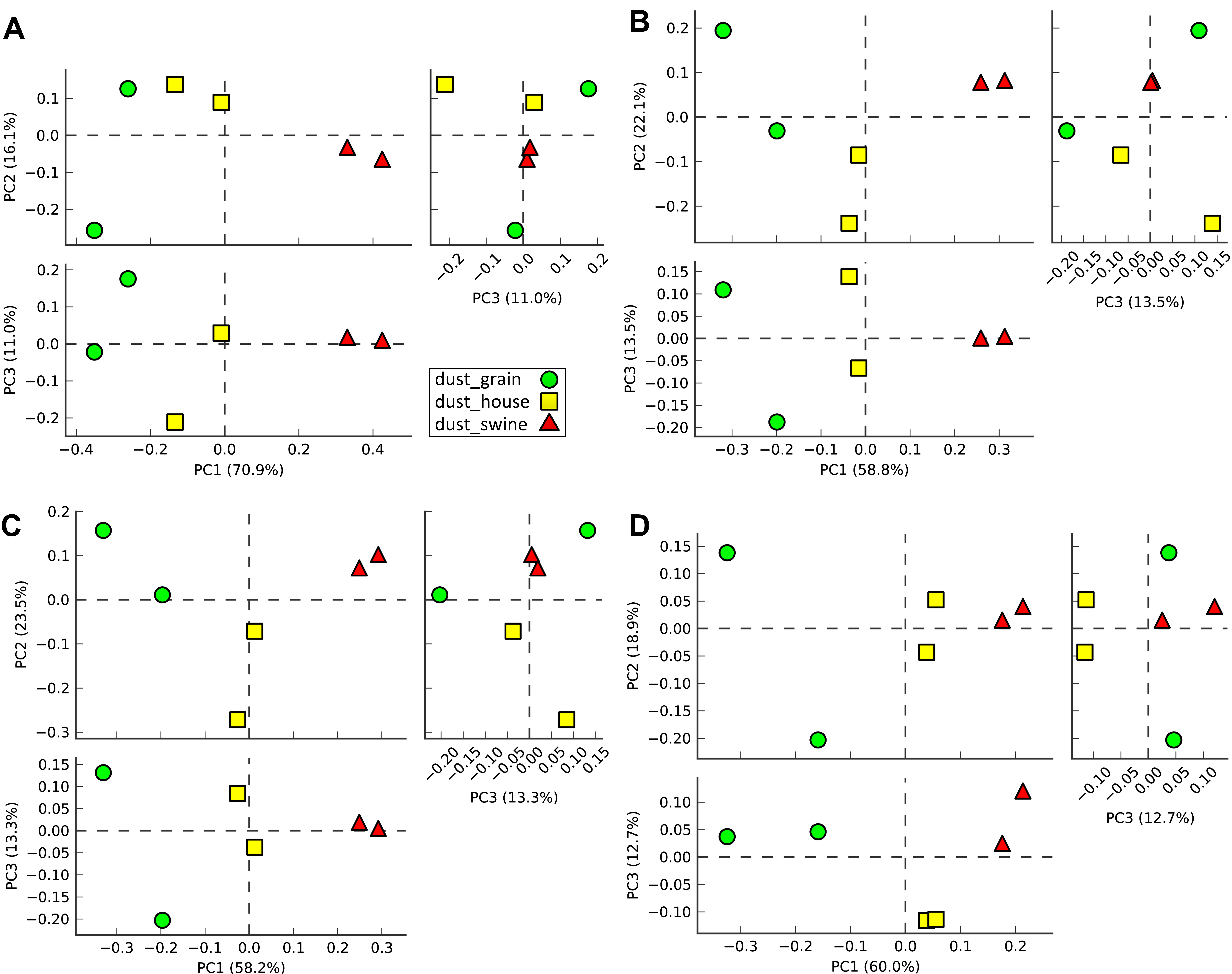

Supplement: Figure S5 — Principal Component Analysis (PCA) of shotgun metagenomic reads from swine facility dust, grain elevator dust, household dust without pets and swine feces. PCA was performed in STAMP using MG-RAST different taxa-level organism abundance profiles (A. Phylum; B. Class; C. Order; D. Family) that were derived from two swine facility dust samples, two grain elevator dust samples, and two household dust samples without pets. “Filtered” reads from the swine confinement facility dust and the household dust datasets were used in the analyses. Each symbol represents one sample. • Grain elevator dust (green); ▪ Household dust without pets (“filtered”, yellow); ▴ Swine confinement facility dust (“filtered”, red). (TIF) [file pone.0095578.s005.tif]
