# Supplementary material for: Shotgun Pyrosequencing Metagenomic Analyses of Dusts from Swine Confinement and Grain Facilities
Source: PLoS One. 2014 Apr 18;9(4):e95578. doi: 10.1371/journal.pone.0095578 (PMC3991671; doi:10.1371/journal.pone.0095578)
Supplement: Table S1 — (DOCX) [file pone.0095578.s006.docx]

**SupplementaryTable ST1.** Filtering of post-QC shotgun metagenomic read datasets with Human and Swine BLASTn alignments.

| **Sample** | **Alignment Database** | **Total Reads** | **Reads With Hit**  **(No.)** | **Reads With Hit**  **(%)** |
| --- | --- | --- | --- | --- |
| Swine dust 1 | *S. scrofa*_unmasked | 26,880 | 3,449 | 13% |
|  | *H. sapiens*_unmasked | 26,880 | 2,682 | 10% |
|  | [*S. scrofa* + *H. sapiens* best hits] | 26,880 | 3,594 | 13% |
| [*S. scrofa* + *H. sapiens* best hits] | Bacterial complete+draft | 3,594 | 233 | 6% |
| Swine dust 2 | *S. scrofa*_unmasked | 18,613 | 4,648 | 25% |
|  | *H. sapiens*_unmasked | 18,613 | 3,548 | 19% |
|  | [*S. scrofa* + *H. sapiens* best hits] | 18,613 | 4,830 | 26% |
| [*S. scrofa* + *H. sapiens* best hits] | Bacterial complete+draft | 4,830 | 146 | 3% |
| Swine feces 1 SRX065862 | *S. scrofa*_unmasked | 20,000 | 402 | 2% |
|  | *H. sapiens*_unmasked | 20,000 | 334 | 2% |
| Swine feces 2 SRX065867 | *S. scrofa*_unmasked | 20,000 | 305 | 2% |
|  | *H. sapiens*_unmasked | 20,000 | 255 | 1% |
| Swine feces 3 SRX065871 | *S. scrofa*_unmasked | 20,000 | 525 | 3% |
|  | *H. sapiens*_unmasked | 20,000 | 464 | 2% |
| Grain dust 1 | *H. sapiens*_unmasked | 38,317 | 753 | 2% |
| Grain dust 2 | *H. sapiens*_unmasked | 21,027 | 381 | 2% |
| Household dust 1 | *H. sapiens*_unmasked | 4,668 | 3,777 | 81% |
| [*H. sapiens* best hits] | Bacterial complete+draft | 3,777 | 17 | 0.4% |
| Household dust 2 | *H. sapiens*_unmasked | 41,571 | 17,942 | 43% |
| [*H. sapiens* best hits] | Bacterial complete+draft | 17,942 | 208 | 1% |
