# Supplementary material for: Shotgun Pyrosequencing Metagenomic Analyses of Dusts from Swine Confinement and Grain Facilities
Source: PLoS One. 2014 Apr 18;9(4):e95578. doi: 10.1371/journal.pone.0095578 (PMC3991671; doi:10.1371/journal.pone.0095578)
Supplement: Table S2 — (DOCX) [file pone.0095578.s007.docx]

**Supplementary Table ST2.** Viral classification of metagenomic reads from swine confinement facility dust, grain elevator dust and household dust without pets.

| **Sample** | **Family** | **Genus** | **Relative Abundance (%)** |
| --- | --- | --- | --- |
| Swine Confinement Dust | *Siphoviridae* | unclassified | 0.152 |
|  | *Myoviridae* | unclassified | 0.059 |
|  | *Siphoviridae* | SPβ-like | 0.017 |
|  | *Myoviridae* | SPO-1-like | 0.008 |
|  | *Myoviridae* | T4-like | 0.008 |
|  | *Podoviridae* | N4-like | 0.008 |
|  | *Podoviridae* | unclassified | 0.008 |
| Grain Elevator Dust | *Myoviridae* | unclassified | 0.009 |
|  | *Siphoviridae* | unclassified | 0.006 |
|  | *Podoviridae* | N4-like | 0.005 |
|  | *Podoviridae* | Epsilon15-like | 0.002 |
|  | *Siphoviridae* | Lambda-like | 0.002 |
|  | *Myoviridae* | T4-like | 0.002 |
|  | *Podoviridae* | T7-like | 0.002 |
|  | *Myoviridae* | P2-like | 0.0005 |
| Household Dust | *Siphoviridae* | unclassified | 0.033 |
|  | *Papillomaviridae* | β-papillomavirus | 0.012 |
|  | *Caulimoviridae* | Cavemovirus | 0.003 |
